# The Role of Maternal Immune Activation in the Pathogenesis of Autism: A Review of the Evidence, Proposed Mechanisms and Implications for Treatment

**DOI:** 10.3390/ijms222111516

**Published:** 2021-10-26

**Authors:** Aleksandra Zawadzka, Magdalena Cieślik, Agata Adamczyk

**Affiliations:** Department of Cellular Signalling, Mossakowski Medical Research Institute, Polish Academy of Sciences, Pawińskiego 5, 02-106 Warsaw, Poland; azawadzka@imdik.pan.pl

**Keywords:** autism, maternal immune activation (MIA), inflammation, cytokines, therapeutic strategy

## Abstract

Autism spectrum disorder (ASD) is a neurodevelopmental disease that is characterized by a deficit in social interactions and communication, as well as repetitive and restrictive behaviors. Increasing lines of evidence suggest an important role for immune dysregulation and/or inflammation in the development of ASD. Recently, a relationship between inflammation, oxidative stress, and mitochondrial dysfunction has been reported in the brain tissue of individuals with ASD. Some recent studies have also reported oxidative stress and mitochondrial abnormalities in animal models of maternal immune activation (MIA). This review is focused on the hypothesis that MIA induces microglial activation, oxidative stress, and mitochondrial dysfunction, a deleterious trio in the brain that can lead to neuroinflammation and neurodevelopmental pathologies in offspring. Infection during pregnancy activates the mother’s immune system to release proinflammatory cytokines, such as IL-6, TNF-α, and others. Furthermore, these cytokines can directly cross the placenta and enter the fetal circulation, or activate resident immune cells, resulting in an increased production of proinflammatory cytokines, including IL-6. Proinflammatory cytokines that cross the blood–brain barrier (BBB) may initiate a neuroinflammation cascade, starting with the activation of the microglia. Inflammatory processes induce oxidative stress and mitochondrial dysfunction that, in turn, may exacerbate oxidative stress in a self-perpetuating vicious cycle that can lead to downstream abnormalities in brain development and behavior.

## 1. Introduction

Autism spectrum disorder (ASD) is defined as a neurodevelopmental illness, the diagnosis of which is based on two fundamental components: deficiencies in social communication, and restricted repetitive behaviors. Deficits in social communication and social interaction include the lack of social–emotional reciprocity (e.g., problems with normal conversations and difficulty in initiating and responding to social interactions), trouble with nonverbal communicative behaviors (e.g., eye contact, body language, and gestures), and deficits in establishing and sustaining relationships (e.g., problems with imaginative play and making friends, as well as a lack of interest in other children). Restricted repetitive patterns of behavior or interests can manifest as stereotypical movements or speech (e.g., lining up toys and repetitive phrases), inflexibility of behavior (e.g., extreme difficulty accepting change or transition and specific rituals associated with regular activities), fixated interests (e.g., strong attachment to objects, sometimes unusual objects, and restricted interests), and atypical responses to sensory stimuli (e.g., hyperreactivity or hyporeactivity, such as intense sensitivity to sound or touch or indifference to pain and temperature) [1]. The first symptoms start to manifest in early childhood, causing impairment in many areas of societal functioning. What is important, however, is that individuals on the spectrum can exhibit different types and severities of symptoms. 

The Autism and Developmental Disabilities Monitoring (ADDM) Network, which analyzes the prevalence of ASD in the U.S. among children aged eight years old, reported that about 1 in 54 children had been identified with ASD in the U.S. in 2016. Importantly, a dramatic increase in the number of reported cases has been observed in the last 16 years, from 67 out of 10,000 (0.67%) in 2000, to 185 out of 10,000 (1.85%) in 2016 [2,3]. Such an increase may be the result of increased public awareness and changes in the diagnostic standards, but an actual increase in risk factors is also a possibility. ASD is diagnosed in all racial, ethnic, and socioeconomic groups, but it has a stark difference in the rate of diagnoses when it comes to sex: ASD is four times more common in males than in females [3]. However, some research indicates that this number might not be accurate. Current theories suggest the existence of a female phenotype of ASD, which has a different presentation from that of the male phenotype, making it harder for females to receive a proper diagnosis [4]. The most interesting trait in the female phenotype of ASD is “camouflaging”. The camouflage hypothesis states that females with ASD are much better than males with ASD at imitating behavior that is considered socially acceptable [5]. Even though females with ASD seem to be functioning well in society as a result of the coping mechanisms they develop, it has been reported that camouflaging comes with the cost of exhaustion, stress, and anxiety. Females and males with ASD deserve a proper diagnosis and support without regard to their sex. 

Regardless of the many theories on the etiology of ASD, the exact cause remains elusive. Currently, ASD is believed to result from the interaction between genetic and environmental factors [6]. Twin studies have shown a high concordance among monozygotic (MZ) twins that is much lower in dizygotic (DZ) twins, demonstrating that ASD has a strong genetic link [7]. It appears that de novo and inherited genetic variants are causal in 10–30% of patients with ASD. Some gene mutations that increase the risk of ASD have been known for a while, such as mutations in the *TSC1*/*TSC2* (tuberous sclerosis complex) or *FMR1* (fragile X mental retardation 1) genes, and new ones are being discovered (e.g., *CHD8*, chromodomain helicase DNA-binding protein 8; *DYRK1A*, dual-specificity tyrosine-(Y)-phosphorylation-regulated kinase 1A; and *SCN2A,* sodium voltage-gated channel alpha subunit 2) [8]. Till date, thousands of genes, copy number variants (CNV), and de novo mutations (DNMs) have been associated with ASD, but no risk loci that are common in all ASD cases have been found. As a result, in addition to genetic research, a great deal of research has been dedicated to potential environmental factors, including chemicals, such as valproic acid (VPA), a well-known drug used in the treatment of epilepsy and migraines, the use of which during pregnancy can severely impact a child’s neurodevelopment [9]. Additional factors, such as exposure to air pollution and paternal age at conception, may increase the likelihood of ASD in offspring [10,11]. Considering the growing trend in the Western world of having children later in life, this research is vital in our understanding of the risk factors for ASD. Maternal diabetes may also be a risk factor for ASD. It has been shown that the risk of ASD in offspring was elevated in mothers with type 1 or type 2 diabetes and gestational diabetes mellitus, as compared to healthy mothers [12]. Finally, the role of maternal immune activation (MIA) during pregnancy in ASD development has also shown potential as a risk factor [13]. In addition, epidemiological studies have shown that there is an association between maternal infection during pregnancy and central nervous system (CNS) diseases with neurodevelopmental origins in offspring. Exposure to different pathogens, such as the rubella virus, *Cytomegalovirus*, or *Toxoplasma gondii,* have also been linked to a higher risk of neurodevelopmental and neuropsychiatric disorders in children, including ASD, schizophrenia, bipolar disorder, major depressive disorder, epilepsy, and cerebral palsy [14,15,16,17]. A connection between maternal infection and a child’s ASD diagnosis was first proposed after the U.S. rubella epidemic of the 1960s. Children prenatally (especially in the first trimester) exposed to rubella have often been born with so-called congenital rubella syndrome (CRS), and as much as 8–13% of these children were later diagnosed with ASD [14,18]. Interestingly, the activation of the maternal immune system, rather than the infection itself, seems to be related to the increased risk of ASD in offspring [19]. A meta-analysis by Jiang et al. indicated that maternal infection during pregnancy, whether it was bacterial, viral, or otherwise, was associated with a 12% increase in the risk of ASD in offspring [20]. In the same meta-analysis, the risk increased significantly if the infections occurred in the first or second trimester, but not for infections occurring in the third trimester. Moreover, fever, a common symptom of inflammation, has been associated with an increased ASD risk in offspring, especially if the fever occurred in the second trimester, which suggests that the timing of the MIA is relevant [21]. An analysis of data obtained from the Danish Medical Birth Register revealed that the admission of pregnant people to the hospital due to viral infections in the first trimester, or bacterial infections in the second trimester, was associated with a later diagnosis of ASD in the offspring [22,23]. Epidemiological observations are further supported by research on MIA in animal models, including rodents and nonhuman primates. The most popular way of inducing prenatal inflammation is with the administration of either double-stranded RNA polyinosinic–polycytidylic acid (poly (I:C), to mimic viral infection), or lipopolysaccharide (LPS, to mimic bacterial infection) [24]. ASD symptoms can also be induced by prenatal VPA injection [25] or IL-6 administration [26]. Our recent results, with rodent models, on the maternal infection risk factor induced by LPS have revealed that the offspring display features of autism, as well as immune-related disruptions in the brain and periphery [27,28]. 

In this review, we focus on the possible links between MIA, inflammation, and the changes in the brain of the offspring that develop into ASD symptoms. We will analyze both animal and human studies completed in the last few years to summarize the existing knowledge about the role of MIA in the pathogenesis of ASD and the prospective for potential therapies.

## 2. Proinflammatory Cytokines as the Link between Maternal Inflammation and Autism Development in Offspring

Pregnancy is considered an immunologically unique state, characterized by the delicate balance between the toleration of a semi-allogeneic fetus, and the protection of both mother and offspring against pathogens. The maternal immune system is highly engaged in the successful progression of pregnancy from implantation to delivery [29]. The most important immune cells found in the maternal–fetal interface are uterine natural killer (uNK) cells, macrophages, T cells, B cells, and dendritic cells (DC) [30,31]. For pregnancy to advance, cytokines need to be secreted in large amounts by both the mother and the developing fetus. After fertilization, implantation of the newly forming embryo takes place: the blastocyst attaches to the endometrial epithelial cells and then invades the endometrial stroma. This is followed by the decidualization of the endometrium and, later, the proliferation of the trophoblast, which is responsible for establishing the blood supply for the embryo [29]. The combination of these processes triggers an inflammatory response that is controlled by cytokines, including leukemia inhibitory factor (LIF), IL-6, and IL-1β [32]. LIF belongs to the IL-6 family and, during implantation, it is secreted by the endometrial cells and orchestrates trophoblast cell adhesion, decidualization and, later, placental development [32,33]. The upregulation of IL-6 and IL-1β during implantation is an evolutionarily conserved mechanism [34]. During implantation, IL-6 is produced by the endometrial epithelium and stromal cells, as well as by the embryo [29]. Later, this cytokine takes part in placental formation, early gestation and, finally, partition [35]. The highest expression of maternal IL-1β is observed in the first trimester of pregnancy [32]. Both IL-1β and interleukin-1 receptor antagonists (IL-1Ra) play important roles in the implantation and development of the placenta. In vitro studies demonstrated that trophoblasts treated with IL-1β release human chorionic gonadotropin (hCG), a key player in early pregnancy development [36]. IL-1β was also recognized as a mediator of contractions during labor [34,36]. In addition, both IL-6 and IL-1β are involved in the development of the CNS, modulating neuronal and glial cell growth and survival [37,38]. 

As was mentioned before, successful pregnancy would not be possible without the tolerance of the maternal immune system for the developing fetus. Such tolerance is achieved because of a specific property of the extravillous trophoblast (EVT) cells. EVT cells do not express the classical MHC class I antigens, which in humans are known as human leukocyte antigens (HLA), except HLA-C, and instead express nonclassical MHC class I antigens: HLA-E, HLA-F, and HLA-G [39]. EVT cells are in direct contact with maternal uNK cells and macrophages, promoting maternal–fetal immune tolerance via interactions between HLA ligands and NK receptors [40]. 

The exact molecular pathways that lead from MIA to ASD, and other neurodevelopmental disorders, are not fully understood. However, a fair amount of research indicates that cytokines and chemokines may play an important role in the process, especially since both are crucial for a successful pregnancy [34]. It appears that viral or bacterial maternal infections can alter the aforementioned state of balance and trigger acute immune activation and the transient upregulation of proinflammatory cytokines that may affect the development of the fetus. Therefore, it is suggested that the MIA-induced alterations of the maternal and fetal cytokine profiles play a key role in ASD development [41]. In this review, we will focus on IL-6 and IL-1β, as they are essential in physiological pregnancy and involved in ASD development [42], as well as on IL-17, which has been considered especially influential in MIA-induced autism [43] but is also necessary for the maintenance of a successful pregnancy [44]. 

### 2.1. Interleukin-6

IL-6 has been excessively studied in the context of MIA, as it appears to be a key mediator of the effects of MIA [45]. Although this cytokine is upregulated in utero during normal implantation [46], an excess of IL-6 can alter fetal development [35,47]. An elevated level of IL-6 was observed in the frontal cortex and cerebrospinal fluid (CSF) [48], as well as in the cerebellum [49], of autistic children. Moreover, research on animal models has shown an increase of IL-6 levels in the fetal brain shortly after the induction of inflammation in pregnant mothers [50,51,52].

IL-6 activation in the placenta during MIA has been associated with impaired behavior in the offspring [45]. In an MIA rodent model, the elevated levels of IL-6 in the placenta were of maternal origin [53]. IL-6 can access the prenatal environment during MIA by more than one path. First, IL-6 may indirectly influence fetal development through a JAK/STAT3 signaling mechanism [53]. When the maternal immune system is activated, causing the elevation of the IL-6 levels in maternal blood and, therefore, in the placenta, IL-6 activates resident immune cells in the decidua, resulting in IL-6 production in situ. Thereafter, IL-6 binds to its receptor complex at the cell surface, which contains either one membrane-associated subunit (IL-6R), or a soluble form of IL-6R (sIL-6R) and two gp130 subunits [54,55], and activates the JAK/STAT3 pathway. The activation of JAK leads to the recruitment and the phosphorylation of STAT3 and its subsequent dimerization. Later, this newly created complex translocates to the nucleus and regulates gene transcription, along with other genes responsible for the proinflammatory response [42,56]. Hsiao and Patterson demonstrated that maternal IL-6 activates JAK/STAT3 in a fetal part of the placenta, specifically in its spongiotrophoblast layer. As a result, they observed an increased expression of acute-phase genes, including SOCS3 [53]. Moreover, the authors demonstrated that IL-6 caused the downregulation of placental growth hormone (GH) production and signaling, which, in turn, led to the reduced expression of insulin-growth factor 1 (IGF-1) [53]. Both of these hormones are crucial for proper fetal development [41], and IGF-1 has been associated with different neuropsychiatric diseases, including ASD [57]. Wu et al. demonstrated that the activation of IL-6 in the placenta is required in order for MIA-induced abnormalities in the fetal brain to occur. Using knockout mice lacking IL-6Ra specifically in the placental trophoblasts, they demonstrated that the lack of IL-6 signaling in the trophoblasts prevented MIA-induced inflammatory responses, both in the placenta and the fetal brain [58]. Second, it has been suggested that maternal IL-6 can cross the placenta, both in humans [59] and rodents [60]. Given the immaturity of the blood–brain barrier (BBB) at this time [61], we can hypothesize that maternal IL-6 may be able to act directly on the developing fetal brain by inducing the synthesis of fetal IL-6 and other proinflammatory cytokines, possibly through the aforementioned JAK/STAT3 pathway. Studies on transgenic mice with chronic elevated IL-6 expression in their CNSs showed signs of astrogliosis and altered neuronal function in their brains, especially in the hippocampus and cerebellum, that was accompanied by changes in their behavior [61]. Moreover, elevated IL-6 levels have been associated with the alteration of excitatory and inhibitory synaptic formations and functions in the brains of animal models [62]. With that in mind, it may be logical to conclude that excessive amounts of IL-6 during the delicate stages of brain development may impact the functioning of the CNS. However, it is important to emphasize that there is still uncertainty concerning the origin of the IL-6 that has such a critical influence on the fetal brain during MIA, and whether it originates in the mother, the placenta, the fetal brain, or the fetal periphery. 

A single injection of IL-6 in pregnant mice caused behavioral changes, including impaired social interactions in the offspring [53,63]. Moreover, prenatal exposure to this cytokine resulted in impaired spatial learning [47]. Importantly, in the MIA model induced by poly (I:C) injection, the simultaneous administration of an IL-6-neutralizing antibody, along with the poly (I:C) injection, was enough to attenuate behavioral changes [63,64]. These results demonstrate the importance of IL-6 in MIA-induced neurodevelopmental disorders. 

### 2.2. Interleukin-17

IL-17A (commonly called IL-17), is a proinflammatory cytokine expressed by TH17 cells (IL-17A^+^CD4^+^ T), the differentiation of which depends on a whole set of transcription factors, including STAT3 and the retinoic acid receptor-related orphan nuclear receptor, γt (RORγt) [65]. Using an MIA animal model, Choi et al. demonstrated that maternal CD4^+^ T cells expressing RORγt are essential for the ASD-like phenotype to occur in offspring [64]. Both of these receptors are regulated by IL-6 and, therefore, an increase in the level of IL-6 in the placenta and/or the fetal brain leads to the upregulation of IL-17A, which is known to promote the production of a variety of cytokines, including IL-6, IL-1β, and TNF-α, as well as a granulocyte-macrophage colony-stimulating factor (GM-CSF), and a granulocyte colony-stimulating factor (G-CSF) [43]. Elevated levels of IL-17A were observed in the serum of ASD children [66] and in the neutrophils of ASD patients [67], where it was accompanied by the upregulation of phospho-nuclear factor-kappa B (p-NFκB), IL-6, and NADPH oxidase 2 (NOX2)/ROS, which suggests that this cytokine plays a substantial role in the modulation of inflammation. High levels of IL-17A have been associated with several autoimmune diseases, and they were also linked to brain pathology in disorders such as epilepsy, ischemia, and multiple sclerosis (MS) [43]. 

**Figure 1:**
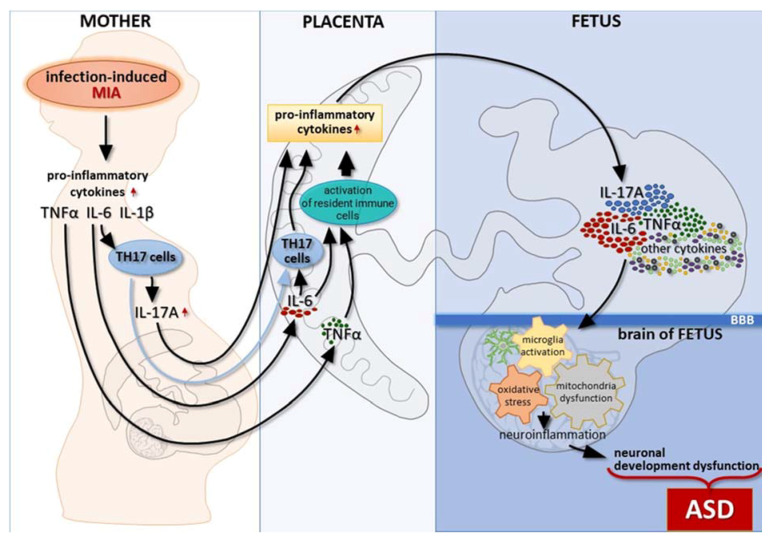
Immunological changes in the placenta and fetal brain in response to systemic inflammation during pregnancy resulting in neuronal development dysfunction in offspring. Infection during pregnancy activates the mother’s immune system, which releases proinflammatory cytokines, such as IL-6, Il-1β, TNF-α, and others. The elevated level of IL-6 leads to the activation of maternal TH17 cells. As a consequence, IL-17A is released and, together with IL-6 and TNF-α, may reach the placenta, where they additionally activate resident immune cells, resulting in an increased production of proinflammatory cytokines, including IL-6. Moreover, activated maternal TH17 cells also transmigrate through the placenta and enhance cytokine production, which affects placenta function and causes damage. This allows the cytokines to pass through to the developing fetus and enhance the production of fetus-derived cytokines. Then, proinflammatory cytokines cross the BBB and initiate a neuroinflammation cascade. This leads to the abnormal development of neurons, as well as alterations in synaptic transmission, which may further lead to the development of ASD in subsequent stages of growth.

A few mechanisms have been proposed for the way by which IL-17A can find the fetal brain, although the exact process has not been confirmed. MIA-induced IL-6 expression in the placenta is followed by the differentiation of maternal TH17 cells and the increased secretion of IL-17A, which appears to be capable of crossing the placental barrier [43]. In addition, the transmigration of activated maternal TH17 cells across the placenta [68] allows the secretion of IL-17A directly into the prenatal environment (Figure 1). IL-17A may also be induced in the fetal brain by maternal IL-6 that has crossed the placental barrier. After secretion, it could influence cells that express its receptor in the developing CNS. 

The exact molecular pathways that lead from IL-17A to MIA-triggered abnormalities are not yet known, though there are a few possibilities worth consideration. After the binding of IL-17A, interleukin 17 receptor A (IL-17RA) joins with the IL-17RC subunit to form a receptor complex [69]. Afterward, adaptor molecule Act1, a protein critical for the mediation of IL-17A signaling, is recruited [70]. Act1 (sometimes referred to as CIKS) is known to be an NF-κB activator [71], allowing it (in a cascade of events) to rapidly translocate into the nucleus, which is followed by inflammatory gene transcription. It is also required for the IL-17-induced expression of the inhibitor of nuclear factor-kappa B zeta-(IκB-ζ) [72], a member of the NF-κB transcription factor family. IL-17 induces IκB-ζ mRNA and protein expression, which allows for the IκB-ζ-dependent activation of various IL-17 target genes in cooperation with NF-κB [73]. NF-κB plays a role in controlling the growth of neural processes in the developing peripheral nervous system (PNS) and the CNS [74] and, furthermore, increased expression and enriched NF-κB signaling has been found in some studies performed on the peripheral blood samples and postmortem brains of ASD patients, as well as in studies on animal models [75]. That said, it seems that NF-κB pathways are a promising research area within the context of ASD etiology. IL-17 also activates the mitogen-activated protein kinase (MAPK) pathways, which include the extracellular signal-regulated kinase (ERK), p38, and JUN N-terminal kinase (JNK) pathways [43,73]. The ERK/MAPK signaling is known to play a critical role in brain development, as well as in learning, memory, and cognition [76]. Therefore, it may be possible that MIA, through excessive IL-17A expression in the fetal brain, might impact ASD symptoms through the dysregulation of this pathway. Importantly, mutations in ERK-pathway genes have been observed in patients with ASD [77], and the dysregulation of ERK signaling has been observed in animal models and the lymphocytes of ASD patients [43].

Choi et al. proved that maternal IL-17 promoted behavioral abnormalities in MIA offspring when the pretreatment of MIA mothers with IL-17A-neutralizing antibodies prevented the behavioral abnormalities. Moreover, atypical cortical development, which has been observed in MIA offspring, was averted by the administration of anti-IL-17A to the mothers. Finally, the authors showed that IL-17A administration directly into the fetal brain promoted atypical cortical development and ASD-like behavioral abnormalities. These same symptoms did not occur when IL-17A was injected into the brains of knockout mouse fetuses lacking IL-17RA [64]. 

### 2.3. Interleukin-1β

Interleukin-1β (IL-1β) is a proinflammatory cytokine expressed by various types of cells, including macrophages, DCs, monocytes, and microglia [78]. It is first produced in an inactive form as pro-IL-1β, which maturates when processed by caspase-1 [79], and after secretion, it initiates local and systemic inflammation. Increased levels of this cytokine have been found in the blood of patients with ASD [80]. Our group also observed elevated levels of IL-1β in the blood of rats prenatally exposed to MIA [27], whereas others detected excessive amounts of this cytokine in the fetal brain of MIA animals [50,51]. In addition, in monocyte cultures from ASD pediatric patients, an increased expression of IL-1β in response to the stimulation of TLR4 (LPS) was observed, in comparison to the controls [81]. That considered, it is possible that this cytokine takes part in the etiology of MIA-induced FASD. 

It is not clear whether the IL-1β that disrupts fetal brain development after MIA is of maternal or fetal origin. IL-1β does not cross the placenta, but it is capable of crossing the BBB [82]. Its expression, however, is triggered not only by TLR4 signaling, but also by the presence of other cytokines, including IL-6 [78], IL-17 [83], and IL-1β itself [84]. It is possible that the secretion of fetal IL-1β is being triggered in response to inflammatory processes developing in the placenta and the aforementioned expression of cytokines. This requires further investigation. Nevertheless, IL-1β is known to affect the developing brain. Crampton et al. tested the influence of this cytokine on neural progenitor cells isolated from the developing rat ventral mesencephalon. The authors observed that IL-1β caused the inhibition of neural progenitor cell proliferation, accompanied by differentiation, where it promoted gliogenesis rather than neurogenesis. This effect was mediated by interleukin 1 receptor type I (IL-1R1) and MAPK pathway signaling [85]. IL-1R1 can also mediate signals via TNF, receptor-associated factor 6 (TRAF6), and the IKK complex, which results in the activation of NF-κB and is followed by inflammatory gene transcription [86]. Chudnovets et al. demonstrated changes in the placenta and fetal brain of mice treated with intraperitoneal injections of different doses of IL-1β from embryonic days (E) 14–17. The authors observed increased IL-1β in the placentas, as well as increased p-NF-κB and caspase-1 in the placentas and in the fetal brains of IL-1β-treated animals, but not consistently in the spleens, suggesting the induction of intrinsic IL-1β production. This was accompanied by the placental disruption–distortion of the labyrinth structure, decreased numbers of mononuclear trophoblast giant cells, and reduced proportions of endothelial cells, as compared to the control placentas. Moreover, reduced fetal viability and a reduction in the cortical neuronal morphology in fetal brains were observed [84]. That placental and fetal brain disturbance, resulting from the IL-1β influence, is confirmed by the fact that the coadministration of the IL-1 receptor antagonist (IL-1Ra), together with LPS, to pregnant females, was demonstrated to have protective effects, as compared to the LPS injection alone [87]. Taken together, proinflammatory cytokines increase in the placenta, and then in the fetal brain, in response to systemic inflammation during pregnancy, and may affect neuronal development dysfunction in offspring. Proinflammatory cytokines that cross the BBB might initiate a neuroinflammation cascade as a result of microglia activation (Figure 1).

## 3. Microglial Abnormalities in MIA-Induced Autism Spectrum Disorders

Microglia are the tissue-resident macrophage-like immune cells of the CNS that play a significant role in brain development, maturation, and maintenance, both in embryonic and adult organisms. Interestingly, microglia from the prenatal brain differ from those in the adult brain, not only morphologically, but also in the expression signatures. Moreover, prenatal microglia appear to proliferate more than adult microglia [88]. During embryonic development, the microglia differentiate from the immune progenitors in the fetal yolk sac and are present in the developing CNS around embryonic day 9.5 (E9.5) [89]. In the CNS, they take part in directing the migration of neural precursor cells and influence their differentiation into mature neurons [90]. During later development, microglia interact with the neurons and the astrocytes involved in the synaptic pruning process that permits the elimination of nonfunctional, or weak, synaptic connections [91]. In addition, it has been suggested that the microglia support the oligodendrocyte precursor cells involved in the myelination processes and influence neovascularization in the developing retina [88]. Later in life, microglia are responsible for maintaining homeostasis in the CNS. In physiological conditions, microglia appear to remain in a resting state, but this calmness is misleading, as microglial processes are constantly expanding and contracting, scanning their surroundings, and engaging in multiple interactions with other cells [92]. The situation dramatically changes upon infection or injury. Microglia are rapidly activated to secrete effector molecules, including proinflammatory cytokines (e.g., IL-1β, IL-6, TNF-α, IL-23, and IL-18) and ROS, causing neuroinflammation [88]. On the other hand, microglia are capable of secreting growth factors and anti-inflammatory cytokines that promote tissue repair [93]. Microglia-induced inflammation and degeneration, as well as the general dysfunction of microglia, are important factors in many CNS disorders, including ASD.

Evidence suggests that microglia are activated in the brains of ASD individuals [94]. Research conducted on postmortem brain samples revealed an increased microglial activation in the cerebellum and the white matter of ASD patients, as compared to the controls [48], as well as in the cortical regions [48,95], where it is accompanied by a higher microglial density [96] and increased microglial–neuronal spatial clustering [95], indicating the neuronal recruitment of the microglia. The activation of microglia in the brain can lead to molecular changes that need to be studied, and since it is not possible to conduct microglia research on living patients, animal models are a good alternative. Unfortunately, however, the findings obtained using MIA animal models are not conclusive. While some researchers report increased microglia density [87,97], changes in microglia morphology [98,99,100], or changes in microglia process motility [101], others report findings that are inconclusive [102,103,104,105]. The differences in their results might be caused by the diverse protocols for inducing MIA, the variation of time points and brain regions chosen for analysis, and divergent approaches to the definition of microglial activation [92]. Nevertheless, a few possible pathways leading from the MIA, through the microglia, and finally to neurological alterations, have been proposed. Schaafsma et al. demonstrated that LPS-induced MIA results in long-term changes in microglia responsiveness into adulthood, and that fetal microglia produced proinflammatory cytokines that contribute to neuro-inflammation, and that might ultimately affect brain development [106]. Ozaki et al. reported that MIA caused sustained alterations in the patterns of microglial process motility, and that such motility changes in the offspring were observed as early as gestational day 18 (E18) and were sustained well into adolescence [101]. These results emphasize that changes in microglial functions may affect neuronal function and plasticity, as well as susceptibility to developmental disorders in the offspring. A growing body of evidence indicates that proper microglial–neuronal interactions are fundamental for brain development and homeostasis, and that MIA may be one of the factors affecting these interactions. The crucial regulatory systems for microglial–neuronal crosstalk are the CX3CL1-CX3CR1 and the CD200-CD200R, with CX3CL1 (also known as fractalkine) localized on the neurons and the only ligand to the CX3CR1 receptor that can be found on microglial cells [107]. The interaction between the two is involved in the maturation and plasticity of synapses, as well as in the regulation of the functioning of the neuronal network and the immune processes in the CNS [108]. In vitro studies on microglial cultures activated with LPS have demonstrated that treatment with CX3CL1 reduced the production of inflammatory mediators, such as NO, IL-6, TNFα, and IL-1β [109], and it maintained the microglia in a quiescent state [110]. CD200 is a surface glycoprotein expressed on multiple cell types, including neurons [111], whereas CD200R is expressed by cells of myeloid lineage in the CNS, which are predominantly microglia [112]. The binding of CD200 by CD200R induces a signaling cascade that blocks the proinflammatory response of the myeloid cells. Any disturbance in the CD200–CD200R signaling results in microglial activation and can lead to neuroinflammation [113]. Recent studies have shown that MIA provoked by LPS or poly (I:C) leads to microglial–neuronal communication alterations in young male rat offspring through the modulation of CX3CL1–CX3CR1 and/or CD200–CD200R signaling [114]. Altered CX3CL1-CX3CR1 or CD200-CD200R pathways, provoked by MIA, may be involved in the disturbance of bidirectional communication between the neurons and the microglia, as well as in the etiology of neurodevelopmental disorders, including autism. In conclusion, the MIA-provoked alterations of the microglial processes in the brains of offspring may contribute to the underlying pathophysiological mechanisms linking maternal immune activation to subsequent risks for ASD. 

## 4. Oxidative Stress in ASD Individuals and MIA Models

The process of inflammation is often accompanied by oxidative stress, which can be defined as an imbalance between antioxidants and reactive oxygen species (ROS) levels that can result in cellular and molecular damage [115]. ROS, including superoxide radicals (O_2_^•−^), hydrogen peroxide (H_2_O_2_), hydroxyl radicals (^•^OH), and singlet oxygen (^1^O_2_), are byproducts mainly of mitochondrial metabolism during oxidative phosphorylation [116]. On one hand, moderate concentrations of ROS are beneficial in physiological conditions, serving as signaling molecules in redox signaling [117], and taking part in the host defense against pathogens in an oxidative burst [118]. On the other hand, ROS tend to act as a double-edged sword: in pathology, ROS increase oxidative stress attacks on the double bonds of lipids, proteins, and DNA bases, leading to cellular damage. The brain, with its immense demand for energy and high lipid content, is especially vulnerable to oxidative stress [119].

A growing body of evidence links ASD to increased levels of oxidative stress and lower antioxidant capacity [120,121]. Markers of oxidative stress have also been observed in the postmortem brain samples of ASD patients, along with a decreased antioxidant capacity (GSH/GSSG ratio) and an increase in 3-nitrotyrosine (3-NT, marker of oxidative protein damage), 3-chlorotyrosine (3-CT, marker of inflammation), and 8-oxo-deoxyguanosine (8-oxo-dG, marker of oxidative DNA damage) [122]. In addition, a lower total antioxidative status, and a higher oxidative stress index, including decreased GSH concentration and increased catalase (CAT) activity, as well as higher levels of malondialdehyde (MDA) and protein carbonyl content, have been observed in the blood of ASD children [121,123,124]. Different animal studies found a redox imbalance after prenatal exposure to MIA. After prenatal exposure to LPS, an increase in the GSSG level, and a decrease in the GSH level and the GSH/GSSG ratio, followed by an increase in the level of lipid peroxide, were observed in the brains of MIA rats [27,125]. In an LPS-triggered MIA model, increased oxidative stress and the expression of metalloproteinase in the amniotic fluid and fetal brain were indicated [51]. 

Inflammation and oxidative stress are closely related. The two create what can be characterized as a self-perpetuating vicious cycle. Chronic inflammation is connected to oxidative stress and neurodegeneration, whereas oxidative stress is known to induce inflammation [121]. The presence of proinflammatory cytokines and damage-associated molecular patterns (DAMPs) induces phagocytic cells (such as macrophages in the PNS, and microglia in the CNS) to perform an oxidative burst. The deliberate production of ROS in those cells is stimulated by NOX2 activity [126]. As was mentioned earlier, ROS serve as signaling molecules and impact a variety of molecular pathways, including NF-κB and MAPKs [127], which leads to the upregulation of proinflammatory cytokines, including IL-1β, IL-6, and TNF-α [126]. 

## 5. Mitochondrial Dysfunction in MIA-Evoked Autism Spectrum Disorders

High levels of oxidative stress can be connected with mitochondrial dysfunction. However, these organelles play a major role in ROS production in cells, mostly via the enzymes of the electron transport chain (ETC), especially complex I (NADH dehydrogenase) and complex III (cyt *bc*_1_), but also via several matrix proteins and complexes, including enzymes of the tricarboxylic acid (TCA) cycle (e.g., aconitase, pyruvate dehydrogenase, and α-ketoglutarate dehydrogenase), as well as the inner and outer mitochondrial membrane proteins, such as cytochrome P450 enzymes and monoamine oxidase (MAO) [128]. A high concentration of steady-state O_2_^−^ (~5–10× higher than in the nucleus or the cytosol) makes mitochondria a target for ROS-induced damage [129], which can lead to an exacerbation of ROS production, resulting in the aforementioned vicious cycle [130]. The mitochondria in the CNS are damaged not only by the overproduction of ROS locally, but also by the ROS produced by activated microglia elsewhere [131]. The reciprocal relationship between mitochondria and the immune response has been well documented, as mitochondria can impact the immune response, and vice versa [132]. Deficits in bioenergetics and mitochondrial dysfunction have been reported in ASD patients. About 4% of patients with ASD are diagnosed with classic mitochondrial disease (MD). However, the latest research examining the biomarkers of mitochondrial dysfunction suggest that abnormalities of mitochondrial function could affect a much higher percentage of children with ASD, perhaps up to 80% [133]. Observed mitochondrial disturbances include alterations in the levels of respiratory chain proteins, such as a reduced expression of the genes of complex I, complex III, complex IV, and complex V in a brain-region-specific manner in ASD brains [134], as well as decreases in the protein levels of the subunits of these complexes [135,136]. Alterations of protein levels are reflected in the reduced activity of the ETC complexes, mostly complexes I and V, but also complex III in the frontal cortex of individuals with ASD [137]. Considering that complexes I and III are responsible for a major part of ROS production, and that complex V is directly involved in the conversion of ADP into ATP, dysfunction of the said complexes can both reduce energy production and increase ROS levels, both of which contribute to oxidative stress. These pathological changes are accompanied by alterations in the genes involved with mitochondrial transport, membrane potential, and dynamics (fusion and fission) [136,138]. 

Research performed on the lymphoblastoid cell lines (LCLs) derived from children with ASD confirmed mitochondrial dysfunction, characterized by increased respiration, and accompanied by higher sensitivity to oxidative stress. About one-third of ASD-LCLs showed elevated respiratory rates while, at the same time, being more sensitive to ROS exposure [139]. The same research group, comparing mitochondrial function in the LCLs of ASD patients, their unaffected siblings, and unrelated healthy controls demonstrated that ASD LCLs exhibited significantly higher respiratory rates, glycolysis, and glycolytic reserves, as compared to siblings, as well as greater sensitivity to ROS, when compared to both siblings and the controls, which suggests that the overactivity of mitochondria, and the sensitivity to ROS, can be linked directly with ASD [140]. Pecorelli et al. analyzed dermal fibroblasts collected from ASD patients and discovered a higher level of metabolic activity (e.g., oxygen-consuming ratio), accompanied by higher proton-leak values, in ASD fibroblasts, as compared to the control cells. The ASD cells exhibited increased basal and maximal glycolytic rates, and an increased expression of the subunits of all the mitochondrial complexes. Finally, the mitochondria in ASD fibroblasts had an atypical morphology, which was significantly different from the mitochondria in the control cells [141]. Abnormalities in respiratory function were also indicated in the peripheral blood mononuclear cells (PBMCs) that were derived from the blood samples of ASD children, and demonstrated that ASD patients diagnosed with developmental regression (DR) had a much higher maximal oxygen consumption rate (OCR), as compared to the controls [142], which suggests a direct link between the presence of mitochondrial dysfunction and the severity of the observed ASD symptoms. Although the relationships between mitochondrial dysfunction, oxidative stress, and neuroinflammation have been well documented, the etiology of mitochondrial dysfunction in MIA-induced autism remains unclear. There also has not been much research regarding mitochondrial dysfunction in MIA animal models. Giulivi et al. showed that MIA activation by poly (I:C) at early gestation is associated with mitochondrial changes in the immune cells of adult offspring. They evaluated mitochondrial function in splenocytes derived from the spleens of 12-week-old mice prenatally exposed to poly (I:C), which showed decreased oxygen consumption and deficits in complex I in the MIA animals, as compared to the controls [143]. This suggests that prenatal immune changes following the maternal poly (I:C) administration are likely to imprint long-lasting changes in the bioenergetics of the adult offspring. Our investigations indicated that mitochondrial alterations in the MIA-affected brains of the offspring, which included changes in the ultrastructure of the synaptic mitochondria, were accompanied by the impairment of ETC gene expression and its functioning. We also observed decreased mitochondrial membrane potential, as well as increased generation of ROS [27,28]. 

Mitochondrial dysfunction and mitochondrial ROS (mtROS) production may trigger the activation of proinflammatory signaling pathways and, in particular, induce the activation of NF-κB, HIF, and AP-1, which contribute to the production of proinflammatory cytokines, including IL-1β and IL-6 [144]. However, mitochondria are susceptible to the influence of proinflammatory cytokines and chronic inflammation [145] as well as elevated ROS production [146]. IL-1β and TNF-α disrupt the functioning of mitochondria in human chondrocytes, resulting in mitochondrial DNA (mtDNA) damage, decreased energy production, and enhanced ROS production [147]. Moreover, these cytokines have been connected with ETC inhibition, an increase in the permeability of the mitochondrial membrane, the suppression of pyruvate dehydrogenase activity, and the inhibition of oxidative phosphorylation [148]. In addition, mitochondrial dysfunction can result in the release of DAMPs that may be involved in the activation of proinflammatory processes via multiple mechanisms, and the propagation of mitochondria-dependent inflammation, a process called “mitoflammation”. However, many questions surrounding the molecular functions of mtROS and mitoflammation in MIA-dependent autism development remain.

## 6. Potential Treatment Strategies Targeting Inflammatory Pathways in ASD

Understanding the role of inflammation in the pathophysiology of ASD may provide new insights into the therapeutic strategies for this disorder. The maternal infection risk factor is currently being studied in animal models. The experiments involve the infection of pregnant mice or rats by infecting the mother, or by simply activating its immune system, in the absence of pathogens. The most popular studies have been those that involve the maternal injection of poly (I:C) to provoke an antiviral inflammatory response, or the maternal injection of LPS to provoke an antibacterial inflammatory response. Although these approaches activate different molecular mechanisms, the analyses of the offspring have thus far revealed considerable overlap in behavioral abnormalities. In addition, similar results have been obtained in both the mouse and rat models of MIA. The findings summarized in Table 1 demonstrate that immune-related therapies can prevent the development of abnormal behaviors in the offspring in the poly (I:C) and LPS models. 

A growing body of literature has also examined the anti-inflammatory therapies for autism and related neurodevelopmental disorders. In Table 2, a review of the studies concerning medications, both with primary anti-inflammatory actions and those with additional anti-inflammatory properties besides their primary mechanisms of action, has been presented (in alphabetical order).

Amantadine is an antiviral medication that is widely used in the management of central nervous system disorders. Its anti-inflammatory effect involves the inhibition of the release of proinflammatory factors [170,171,172]. A double-blind placebo-controlled study of amantadine hydrochloride in the treatment of children with ASD indicated a significant improvement in the absolute scores of hyperactivity and inappropriate speech, according to the clinician-rated ABC (Aberrant Behavior Checklist-Community version). Amantadine also improved the CGI score (Clinical Global Impressions), as compared to the placebo [153]. In another double-blind placebo-controlled trial on patients with severe behavioral issues, such as disruptive symptoms related to ASD, amantadine was added to risperidone for treatment. The data showed a significant improvement in hyperactivity and irritability measured by the ABC. In addition, the CGI-I scores showed significant improvement in the amantadine group, as compared to the placebo [154]. An NMDA receptor antagonist drug with anti-inflammatory and neuroprotective properties is memantine [173]. In a randomized controlled trial comparing treatment with risperidone with memantine, and risperidone with a placebo, in children with ASD, significant improvements were reported in irritability, stereotypic behaviors, and hyperactivity, as measured by the ABC [163]. Similarly, Joshi et al., in an open-label study, evaluated the efficacy and tolerability of memantine, which also resulted in a significant improvement in autism severity. The 12-week study on adult patients with ASD found that memantine treatment was associated with improvement in the CGI, SRS (Social Responsiveness Scale), and brief psychiatric rating scale scores, as well as in anxiety and nonverbal communication (which were measured using the Diagnostic Analysis of Nonverbal Accuracy Scale). Moreover, the applied treatment was not associated with any serious adverse effects [164].

As a nonsteroidal anti-inflammatory selective inhibitor of cyclooxygenase-2 (COX-2), celecoxib has been widely used as an adjuvant therapy in several psychiatric disorders. In a randomized double-blind controlled trial on children with ASD, treatment with celecoxib as an adjuvant therapy presented significant improvements in irritability, social withdrawal/lethargy, and stereotypic behaviors [155]. Similar results were obtained in the studies using corticosteroids, a class of steroid hormones that exert anti-inflammatory properties [170]. The effects of corticosteroids, or adrenocorticotrophic hormones (ACTH), in regressive ASD, were investigated in two different case studies on a 6-year-old patient and an 18-month-old patient. The research showed that low-dose therapy with corticosteroids significantly improved spontaneous speech, responsiveness to verbal communications, social relatability, receptiveness, and expressive vocabulary [156,157]. The role of ORG 2766, an ACTH analog, in the improvement of ASD behaviors was analyzed by Buitelaar and colleagues in a series of analyses. In a double-blind crossover study, the authors showed that the four-week ORG 2766 administration significantly improved clinical symptoms (e.g., irritability, stereotypic behaviors, hyperactivity, and excessive speech) as measured by the parent-reported ABC [167]. The positive effects of ORG 2766 were also reported in a second crossover trial when administrated for eight weeks. The authors reported significant improvements in stereotypic behaviors and social interactions (e.g., in play behaviors). Furthermore, the adverse effects were minimal [168]. Corticosteroid therapy was also used in a case study on a 6-year-old boy with regressive ASD, over 28 months. Stefanatos et al. described a child whose language and behavior had regressed at 22 months of age, and in whom a pervasive developmental disorder was later diagnosed. The application of corticosteroid therapy resulted in significant improvements in language, social abilities, and stereotypic behaviors [156]. Corticosteroid therapy was also evaluated by Shenoy and colleagues in a case study of an 18-month-old child with regressive ASD who also developed autoimmune lymphoproliferative syndrome (ALPS). This research showed that, after a month of corticosteroid therapy, the patient started regaining his language and communication abilities. After 26 months of therapy, all the laboratory values returned to normal [157]. According to this data, corticosteroids may have potential as a treatment for ASD-like behavior; however, rigorously controlled trials are still needed [170]. 

In the search for an anti-inflammatory therapy for ASD, the possible protective effect of lenalidomide, the immunomodulatory drug derivative of thalidomide and widely used in the treatment of many hematologic disorders, has been considered [170]. Lenalidomide can alter TNF-α with less toxicity than thalidomide. In the open-label pilot study, lenalidomide was administered to seven patients for 12 weeks. The patients showed significant improvements in ASD symptoms, which was measured by the Childhood Autism Rating Scale (CARS), as well as in expressive language, which was measured by the CGI. However, among the seven children enrolled, two developed a rash and had to withdraw from the study [159]. This study suggests that, if tolerated, lenalidomide may promote improvement in behavioral symptoms. 

Recent data have demonstrated that flavonoids, the polyphenolic compounds widely found in fruits and vegetables, can inhibit regulatory enzymes or transcription factors important in controlling proinflammatory mediators [174]. Luteolin and quercetin are plant-derived flavonoids that show a broad range of effects, including antioxidant, anti-inflammatory, anticancer, and neuroprotective properties [161]. Luteolin is capable of inhibiting proinflammatory cytokine expression, NF-kB signaling, and TLR4 signaling, as well as weakening microglial activation [175]. Therefore, in a series of trials, the flavonoid properties were analyzed for ASD treatment. In an open-label pilot study, Tsilioni et al. analyzed the effects of luteolin and quercetin treatments on 50 children for 26 weeks. A total of 40 children completed the protocol, with significant improvements in adaptive functioning, which was measured using the Vineland Adaptive Behavior Scale (VABS), and in overall behavior, as indicated by the reduction (26.6–34.8%) according to the ABC subscale scores [161]. In an open-label case series of 37 children with ASD, Theoharides et al. evaluated the lutein formulation, NeuroProtek, a mixture of luteolin, quercetin, and rutin that is known for its antioxidant, anti-inflammatory, and neuroprotective properties, as a potential treatment for ASD. The results showed improvements in eye contact and attention to directions (in 50% of the patients), as well as in the retention of learned tasks and social interactions (in 30% and 50% of the patients, respectively), and speaking skills (in 10% of the patients) [162]. Additional blood samples from children who received NeuroProtek for four months showed a significant decrease in the mean serum IL-6 and TNF levels at the end of the treatment period. These effects were strongly associated with children whose behavior improved after NeuroProtek treatment. 

Regarding the effectiveness of anti-inflammatory interventions in ASD, attention has been focused on a group of antibiotics in the tetracycline class, including minocycline, with its well-known anti-inflammatory properties. Pardo et al. investigated the effect of minocycline for six months. The open-label study with 10 patients showed protective changes in the profiles of brain-derived neurotrophic factor (BDNF) in CSF and blood, hepatic growth factor (HGF) in CSF, and CXCL8 (IL-8) in serum. However, minocycline did not have a significant effect on core autism symptoms. It was suggested that the group of children may have been too small to observe clinical improvement [165]. Ghaleiha and et al. analyzed minocycline as an adjunctive therapy to risperidone. In the 10-week randomized placebo-controlled study, minocycline showed a significant improvement in the irritability and hyperactivity/noncompliance scores measured by the ABC. Unfortunately, the treatment did not have any significant effect on lethargy/social withdrawal, stereotypic behavior, and inappropriate speech [166]. Thus far, there has been insufficient evidence to support the use of minocycline in the treatment of the core autism symptoms [170].

The presented studies show that anti-inflammatory therapy for ASD has potential. However, the limited data suggest it may only be effective for a certain subset of individuals with ASD. Large-scale randomized controlled trials are needed to provide robust evidence for its efficacy as a therapeutic treatment.

## 7. Conclusions 

The evidence reviewed in this paper strongly suggests a causal relationship between MIA in early gestation and ASD development in offspring (Figure 1). The injection of a single inflammatory cytokine (e.g., IL-6 or IL-17) is sufficient to induce several ASD-like behaviors in offspring, and MIA may induce alterations in multiple cytokines in the fetal brain within a matter of hours. Prenatal immune challenges and proinflammatory cytokines crossing the fetus’ BBB promote the microglial cells to their proinflammatory phenotype. Inflammatory processes in the brain induce oxidative stress and mitochondrial dysfunction that, in turn, may exacerbate oxidative stress in a self-perpetuating vicious cycle, leading to downstream abnormalities in brain development. Thus, MIA-induced immune dysregulation/inflammation, oxidative stress, and mitochondrial dysfunction in the brain of offspring, as well as the linkage between these abnormalities, could be the primary molecular pathways downstream from maternal infection. However, other molecular mechanisms should be taken into consideration. One possibility is that proinflammatory cytokines lead to long-lasting changes in the expression of other classes of immune molecules, including major histocompatibility complex I (MHCI), and molecules that are known to regulate synapse formation, synaptic plasticity, and synaptic pruning, as well as neural connectivity and function in the brains of offspring. It is also possible that immune signaling may converge upon the mammalian target of rapamycin (mTOR) signaling, which has been shown to be altered in the brains of MIA offspring, as well as in individuals with ASD. In summary, while the molecular mechanisms of MIA as an ASD primer requires further research, there is growing evidence that microglial activation, oxidative stress, and mitochondrial dysfunction in the brain of offspring could mediate the neuropathology and behaviors associated with ASD.

## Figures and Tables

**Table 1:** The summary of the studies on potential therapeutic strategies for the MIA treatment.

Animal Model	Applied Treatment	Results	Reference
Poly(I:C) mice model; 2.5 mg/kg *i.p.* from GD 12 to 16	Anti-IL-6 or anti-IL-1β or anti-IL-6 plus anti-IL-1β antibody co-administered with poly(I:C) from GD 12 to 16	Anti-IL-6 antibody reversed the effect of MIA on impaired social interaction	[149]
Poly(I:C) mice model; 20 mg/kg *i.p.* on GD 12.5	IL-17A antibody administered at GD 14.5	IL-17A antibody and IL-17Ra KO prevented elevation of cytokine levels and reversed effects of MIA on impaired ultrasonic vocalizations (USVs), sociability, and repetitive/compulsive-like behaviors	[64]
Poly(I:C) mice model; 20 mg/kg *i.p*. or IL-6, IFN-γ, 5 µg/kg *i.p*. on GD 12.5 and IL-6 KO	Anti-IL-6, IFN-γ, or IL-1β antibodies co-administered with poly(I:C) or IL-6 on GD 12.5	anti-IL-6, IFN-γ, or IL-1β antibodies reversed the effect of MIA on pre-pulse inhibition (PPI). Anti-IL-6 antibodies prevented the exploratory, anxiety, and social deficits provoked by MIA	[63]
Poly(I:C) mice model; 20 mg/kg *i.p.* on GD 12.5	Ibudilast (anti-inflammatory drug) administered to lactating females *i.p.* for 2 weeks (starting 24 h post-parturition)	Ibudilast prevented increased repetitive/compulsive-like behaviors	[150]
Poly(I:C) mice model; 5 mg/kg *i.p.* on GD 10.5, 12.5, and 14.5	A ketogenic diet (KD) at 5 weeks of offspring age for 3–4 weeks	A KD reversed the effect of MIA on impaired sociability and communication as well as repetitive behaviors	[151]
Poly(I:C) mice model; 20 mg/kg *i.p.* on GD 12.5	Probiotics: *Bacteroides fragilis* (ATCC 9343) administered to offspring every other day for 6 days post-weaning (1 × 1010 CFU)	*B. fragilis* regulated serum metabolomic profiles and prevented anxiety-like behaviors	[152]
Poly(I:C) mice model; 20 mg/kg *i.p.* on GD 12.5	Probiotics sachet children’s formula: *Bifidobacteria (B. bifidum & B. infantis), Lactobacillus helveticus,* and fructooligosaccharides administered to mother from GD 0.5 to PD 21	Oral probiotics prevented increases in the IL-6 and IL-17A levels in both maternal serum and fetal brains, and it prevented the MIA-induced social deficits, repetitive/stereotyped behaviors, and anxiety in adult offspring	[52]

Abbreviations: GD, gestational day; *i.p.*, intraperitoneal; KO, knockout; LPS, lipopolysaccharide; poly(I:C), polyinosinic:polycytidylic acid; and PD, post-natal day.

**Table 2:** Summary of the studies on the role of anti-inflammatory medications in ASD treatment.

Applied Drug	Type of Trial	Participants	Results	Reference
Amantadine	Double-blind placebo-controlled trial	39 patients, age: 5–19 years	Significant improvement in absolute score of hyperactivity and inappropriate speech, as compared to placebo group	[153]
Amantadine plus risperidone	Double-blind placebo-controlled trial	39 patients, age: 4–12 years	Significant improvement in hyperactivity and on CGI	[154]
Celecoxib (a nonsteroidal anti-inflammatory drug, selective inhibitor of cyclooxygenase-2 (COX-2) enzyme) plus risperidone	Double-blind placebo-controlled trial	40 patients, age: 4–12 years	Significantly improved scores in irritability, social withdrawal/lethargy, and stereotypic behavior	[155]
Corticosteroid therapy	Case study	6-year-old patient with autoimmune condition plus ASD	Significantly improved spontaneous speech, responsiveness to verbal communications, social relatedness, and receptive and expressive vocabulary Decreased stereotypical utterances	[156]
Corticosteroid therapy (low dose)	Case study	18-month-old patient	Increased social interaction and improvements in speech, gesturing, nonverbal communication, language expression, and comprehensive subjective improvement	[157]
Imuno^®^ (supplement containing low-molecular-weight chondroitin sulfate, phosphatidylcholine and vitamin D_3_)	Open-label study	3 patients	Improvement in behavioral symptoms	[158]
Lenalidomide (derivative of thalidomide)	Pilot, open-label study	7 patients, age: 6–12 years	Some improvement of behavioral symptoms	[159]
Luteolin plus quercetin	Pilot, open-label study	40 patients, age: 4–10 years	Improvement in Vineland Adaptive Behavior Scale (VABS) scores (10 patients), correlated with a significant decrease in serum IL-6 and TNF levels	[160]
Luteolin, quercetin, plus rutin (NeuroProtek^®^ supplement)	Open-label study	40 patients	Significant improvement in bowel color, form, and habits, as well as in eye contact/attention to directions (50%), retention of learned tasks, social interactions, and speaking skills	[161,162]
Memantine plus Risperidone	Double-blind placebo-controlled study	40 patients, age: 4–12 years	Significant improvement in irritability, stereotypic behavior, and hyperactivity (ABC)	[163]
Memantine	Open-label treatment trial	18 patients, age: 18–50 years	Significant improvement in the severity of core features of autism based on SRS and CGI, and improvement in impaired reading and nonverbal communication	[164]
Minocycline	Pilot pen-label study	10 patients, age: 3–13 years	Changes in profiles of BDNF in CSF and blood, HGF in CSF, and CXCL8 (IL-8) in serum. However, the group of children was too small to observe clinical improvement	[165]
Minocycline plus Risperidone	A randomized double-blind placebo-controlled study	46 patients, age: 3–14 years	Significant improvement of irritability and hyperactivity/noncompliance	[166]
ORG 2766 (analog of adrenocorticotropic hormone—(ACTH)	Placebo-controlled double-blind cross-over trial	14 patients, age: 5–13 years	Significant improvement in irritability, stereotypic behaviors, hyperactivity, and excessive speech	[167]
ORG 2766	Double-blind placebo-controlled cross-over trial	20 patients, age: 5–15 years	Significant improvement in stereotypic behaviors and social interaction (play behavior); adverse effects were minimal	[168]
Spironolactone (an aldosterone antagonist and potassium-sparing diuretic)	Case study	12-year-old boy	Significant improvement in irritability, social withdrawal, stereotypy, hyperactivity, inappropriate speech, and receptive language	[169]
